# Human Papillomavirus and Anal Cancer: Prevalence, Genotype Distribution, and Prognosis Aspects from Midwestern Region of Brazil

**DOI:** 10.1155/2019/6018269

**Published:** 2019-09-18

**Authors:** Larisse Silva Dalla Libera, Keila Patrícia Almeida de Carvalho, Jéssica Enocencio Porto Ramos, Lázara Alyne Oliveira Cabral, Rita de Cassia Goncalves de Alencar, Luísa Lina Villa, Rosane Ribeiro Figueiro Alves, Silvia Helena Rabelo Santos, Megmar Aparecida dos Santos Carneiro, Vera Aparecida Saddi

**Affiliations:** ^1^Universidade Federal de Goiás, Postgraduate Program in Health Sciences, Faculty of Medicine, Goiânia, Goiás, CEP 74605-020, Brazil; ^2^Universidade Federal de Goiás, Institute of Tropical Pathology and Public Health, Goiânia, Goiás, CEP 74605-050, Brazil; ^3^Pontifical Catholic University of Goiás, Postgraduate Program in Environmental Sciences and Health, Goiânia, Goiás, CEP 74605-010, Brazil; ^4^Department of Pathology, Hospital Araújo Jorge, Goiânia, Goiás, CEP 74605-050, Brazil; ^5^Cancer Institute of the State of São Paulo, São Paulo, SP, CEP 01246-000, Brazil

## Abstract

**Background:**

Approximately 90% of all anal cancers are associated with human papillomavirus (HPV), especially high-risk genotypes such as HPVs 16 and 18.

**Objective:**

To investigate the clinical and prognostic aspects of anal cancers associated with the presence, as well as the genotypic distribution of human papillomavirus (HPV).

**Methods:**

A retrospective study carried out over a 10-year period, using clinical and molecular data, with PCR analysis and reverse hybridization (INNO-LIPA kit), in anal cancers. The data analysis was done using descriptive univariate statistics, and the survival curves were made using the Kaplan–Meier and log-rank methods.

**Results:**

Of the 81 formalin-fixed and paraffin-embedded specimens, HPV prevalence was 69% and was significantly higher in squamous cell carcinomas (SCC) than in other anal tumors (*p*=0.0001). Female patients had a higher prevalence of HPV (*p*=0.01). Multiple infections were detected in 14.3% of cases. The most prevalent genotypes were HPVs 16, 33, and 18. The overall survival at 60 months was 44.3%, and the prognostic factors included gender (*p*=0.008) with greater survival for men (52.9%) in comparison to women (29.6%), histological type (*p*=0.01), SCC (54.4%), adenocarcinomas (37.5%), other carcinomas (14.2%), and the presence of distant metastasis (*p*=0.01). Survival was not influenced by the presence of HPV (*p*=0.54).

**Conclusions:**

The association of HPV to anal cancer was found in this study, especially in SCC. However, the presence of HPV did not influence the prognosis of patients with anal cancer.

## 1. Introduction

Approximately 5% of all cancers worldwide are associated with human papillomavirus (HPV), and the proportion of anal cancer attributed to HPV is 90%, with genotypes 16 and 18 found in more than 70% of these cancers [1–5]. Anal cancer is a rare tumor that corresponds to approximately 2% of cancers that affect the gastrointestinal system [6]. About 48,000 new cases of anal cancer are diagnosed every year worldwide, with a peak incidence in the age group between 58 and 64 years [7–9].

In Brazil, data on anal cancer are scarce, but it is estimated to correspond to 1% to 2% of all colorectal cancers [10]. In 2010, 274 deaths from anal cancer were recorded in the country, with 98 cases in men and 176 cases in women [11]. In 2013, 348 deaths were recorded, with 106 in men and 242 in women. In 2014, 1,100 new cases of anal cancer were estimated in Brazil [10]. According to the Population Based Cancer Registry of Goiânia, between 1989 and 2008, a total of 117 cases of anal cancer were diagnosed in the city, but more recent data were not available. A study carried out in Goiânia, a city in the Midwest region of Brazil, described 42 cases of anal cancer, of which 38 were tested for HPV DNA and 76% were positive for the presence of HPV DNA [12].

The most common histological type of anal cancer is squamous cell carcinoma (SCC), followed by adenocarcinoma [2, 6, 13]. HPV can be considered an essential factor for the development of SCC, as well as its precursor lesions such as anal intraepithelial neoplasia (AIN) [14, 15]. Several factors contribute to HPV-induced anal carcinogenicity, such as early onset of sexual activity, sexual practices involving anal intercourse, prior exposure to high-risk HPV genotypes, history of anogenital injury, and other cancers associated with HPV. The major risk groups include men who have sex with men, transsexual women, and individuals carrying human immunodeficiency virus (HIV) [14–18].

The role of HPV in the prognosis of anal carcinomas is poorly understood, as well as its influence on the clinical aspects of this tumor [14–16]. Although patients diagnosed with primary cancers associated with HPV respond well to treatment, there is a risk for a second exposure or relapse of HPV infection [14, 18]. Classical aspects of staging, such as tumor size, metastatic lymph node involvement, and distant metastasis, remain the main factors that influence the prognosis of anal carcinomas [6, 19, 20].

The goal of this study was to evaluate the prognostic, sociodemographic, and clinical aspects of individuals with anal cancer associated with the detection and genotypic distribution of HPV, as well as the influence of HPV infection on the prognosis of this tumor.

## 2. Methodology

### 2.1. Type of Study and Sample Selection

This is a retrospective study that investigated HPV prevalence and genotype distribution in a group of anal cancer patients assisted in a cancer reference center from Goiânia, a middle-sized city in the Midwest region of Brazil, during a period of 10 years.

**Figure 1:**
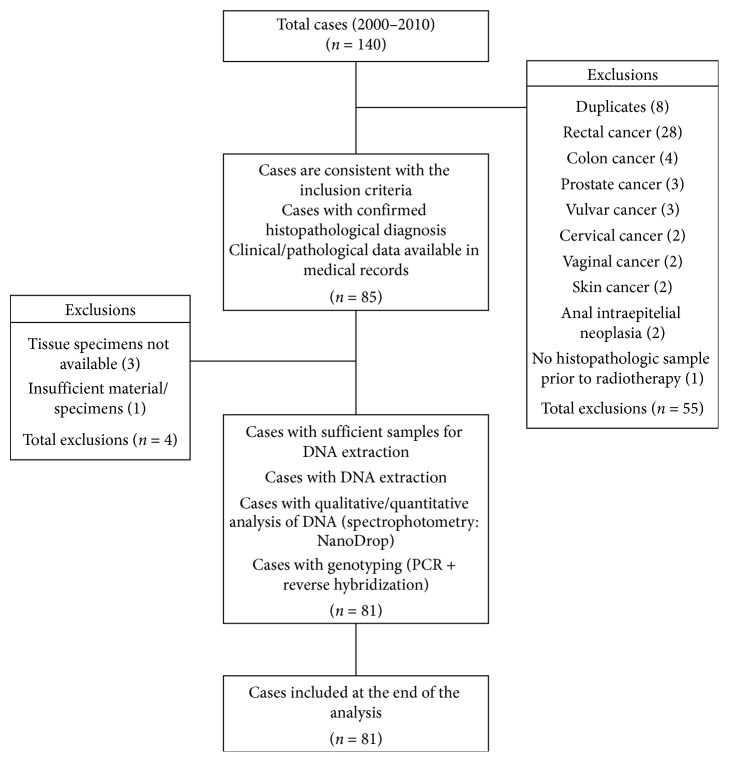
Flowchart of case sample selection.

A sample size calculation was not performed since we aimed to include in the study all the cases that were diagnosed as anal cancer in the Pathology Department of the center. Our reference sample came from the registry of the Pathology Laboratory, so initially, a list of 140 patients diagnosed with anal cancers from 2000 to 2010 was consulted. After pathological/clinical review, 85 cases of anal cancers were considered eligible. A description of the inclusion and exclusion criteria of the cases was presented in Figure 1.

The selected cases were those that presented histopathological diagnoses of anal cancer confirmed by two pathologists, those with clinical data available in the medical records, and those with paraffin blocks available and sufficient for molecular analyses. Cases that were not confirmed as primary anal cancer were excluded. Since we aimed to evaluate five-year overall survival, the retrospective study that was initiated in 2016 considered patients that were diagnosed until 2010.

This study was approved by the Research Ethics Committee of the Association to Combat Cancer in Goiás (CEP/ACCG) under CEP: 272,288.

### 2.2. Preparation of Samples

After selection of the paraffin blocks containing the tumor specimens, each block was serially sectioned with the use of a microtome and the sections packed in properly identified 2 ml sterile microtubes. The microtome knives were changed between samples, and the equipment was cleaned with ethanol for each new block. From each block, slides containing tumor fragments were prepared and stained using hematoxylin and eosin and reviewed by a pathologist. The diagnosis of anal carcinoma was confirmed for each case, based on the classification criteria for tumors from the World Health Organization [21].

### 2.3. DNA Extraction

Viral DNA extraction was performed using the phenol-chloroform-isoamyl alcohol method; the paraffin removal was done with the organic solvent xylol and cell digestion performed with proteinase-K. The final DNA precipitation was done with isopropanol and DNA purification with 70% ethanol. As dewaxing of the sample can lead to tissue loss and consequent degradation of the DNA present in the sample [22], the amount of DNA extracted and its purity were evaluated by spectrophotometry (Thermo Scientific NanoDrop Products). The samples were submitted to polymerase chain reaction (PCR) to amplify the endogenous control, a fragment of the human gene glyceraldehyde-3-phosphate dehydrogenase (GAPDH). Samples negative for endogenous control were re-extracted.

### 2.4. Detection of HPV Genotype

Our study employed the INNO-LiPA HPV Genotyping Extra test (Innogenetics NV, Ghent, Belgium) to detect and genotype HPV DNA, following the manufacturer's instructions. This assay can identify 28 different HPV genotypes, including all known HR-HPV genotypes and probable HR-HPV genotypes (16, 18, 26, 31, 33, 35, 39, 45, 51, 52, 53, 56, 58, 59, 66, 68, 73, and 82), as well as several LR-HPV genotypes (6, 11, 40, 43, 44, 54, and 70) and a number of additional types (69, 71, and 74), based on nested PCR amplification of a fragment (65 base pairs) of the L1 region of the HPV genome. Amplified products were denatured under alkaline conditions and immediately incubated with the test strips in hybridization buffer. The results were visually interpreted by two independent investigators by comparing them with a template provided with the assay. The kit allows simultaneous detection of multiple genotypes in a single sample. Several publications have already proved the performance of the assay in cervical scrapes and in formalin-fixed and paraffin-embedded (FFPE) tissue.

The study employed the INNO-LiPA HPV Genotyping Extra test (Innogenetics NV, Ghent, Belgium) to detect and genotype HPV DNA. This assay is a reverse line hybridization assay validated by several previous studies. The physical state of HPV was not investigated.

In genotyping, only those samples that presented a single genotype of HPV were considered as single infections, and the samples that had more than one HPV genotype were considered multiple infections. In cases of multiple HPV infections with at least one high-risk genotype, the result was considered to be high-risk HPV. In cases that contained only low-risk genotypes, the sample was considered low-risk HPV.

The entire laboratory procedure, from sample handling to HPV detection and genotyping, followed the international standards for HPV testing by the World Health Organization [23].

### 2.5. Statistical Analysis

Sociodemographic and clinical and pathological data were collected on appropriate forms and transferred to spreadsheets, Microsoft Excel, version 2013. The database was digitized by two independent researchers and compared for data verification and database cleanup. The data were transferred to GraphPad Prism version 4.0 and analyzed using descriptive statistics, in order to generate prevalence estimates with respective confidence intervals.

For the age group, the mean and standard deviation were calculated. In order to evaluate the possible associations between the analyzed variables, a univariate analysis was performed considering the level of significance *p* < 0.05 and chi-square test (*χ*^2^). In order to evaluate the associations between the results obtained for HPV detection and the other variables, odds ratios (OR) were calculated with a 95% confidence interval (CI) and significance level of 5%.

The Kaplan–Meier method was used to calculate survival, and the log-rank test was used to compare survival curves against prognostic factors for anal cancer. Death was considered independent of its cause.

The cases included in the study did not present HIV infection status registered in the patient files, and therefore, these data were not used as a prognostic factor.

## 3. Results

### 3.1. Sample Characteristics

Sampling included 81 cases of anal cancer. The characteristics of patients with anal cancer are presented in Table 1.

The majority of the patients were female (63%). Age ranged from 36 to 92 years, and the overall mean age at diagnosis was 61.57 years (±12.73); mean age for women was 62.47 years (±13.01) and for men 60.03 years (±12.30).

Lymph node metastases were reported in 25.9% of patients, and inguinal lymph nodes were the most compromised (data not shown). Distant metastases were described in 8.6% of the group, with the liver and lung being the most affected organs. At the end of 60 months following diagnosis, 55.6% of the patients had reported deaths.

Most of the samples (52%) were diagnosed as anal squamous cell carcinoma, followed by adenocarcinomas (39.5%). The majority of SCC and adenocarcinomas were in T1-T2 stages (*p*=0.01). The other types of anal cancers were in advanced stages, but without lymph node spread. Both SCCs and adenocarcinomas presented cases with distant metastasis.

### 3.2. Prevalence of HPV DNA

The prevalence of HPV DNA for the evaluated group was 69.1%. Only 25 patients were negative for HPV DNA.  Table 2 presents the prevalence of HPV and its association with the social, demographic, clinical, and pathological characteristics investigated. HPV was more prevalent in women than in men (OR 3.18 95% CI 1.19–8.48). The mean age of the group at diagnosis was similar in HPV-negative (63 years ± 11.6) and HPV-positive patients (61 years ± 13.2). HPV was significantly associated with anal SCC (OR 9.51 95% CI 2.96–30.50) (Table 2).

### 3.3. HPV Genotype Distribution

**Figure 2:**
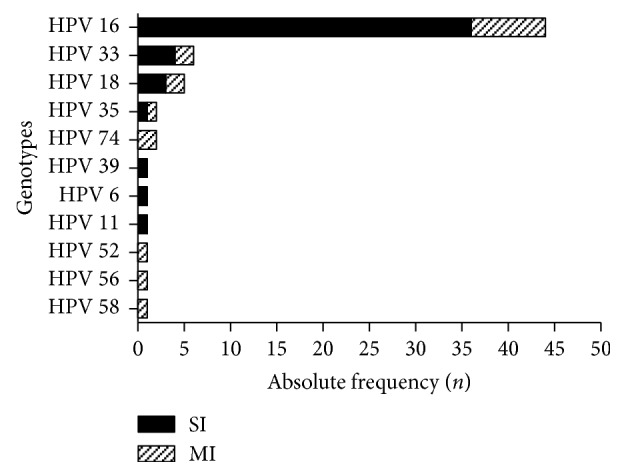
Frequency of 11 HPV genotypes detected in single infection and multiple infections in anal cancers. SI: single infection; MI: multiple infection. Low-risk HPV (LR): 6 and 11. High-risk HPV (HR): 16, 18, 33, 35, 39, 52, 56, 58, and 74.

Of all patients with anal cancer positive for HPV DNA, 85.7% had a single HPV-type infection, while 14.3% had multiple HPV types. The genotypic distribution of HPV and the presence of single and multiple infections of all genotyped samples are described and presented in Figure 2. The most prevalent genotypes in squamous cell carcinomas and anal adenocarcinomas were HPVs 16, 18, and 33. In the other types, the most common were HPVs 16 and 33 (Table 3).

### 3.4. Survival

**Figure 3:**
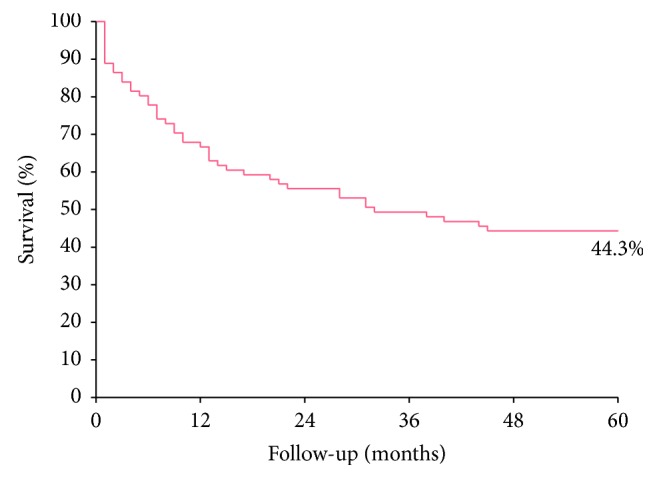
Five-year overall survival for patients with anal cancer (Kaplan–Meier method).

**Figure 4:**
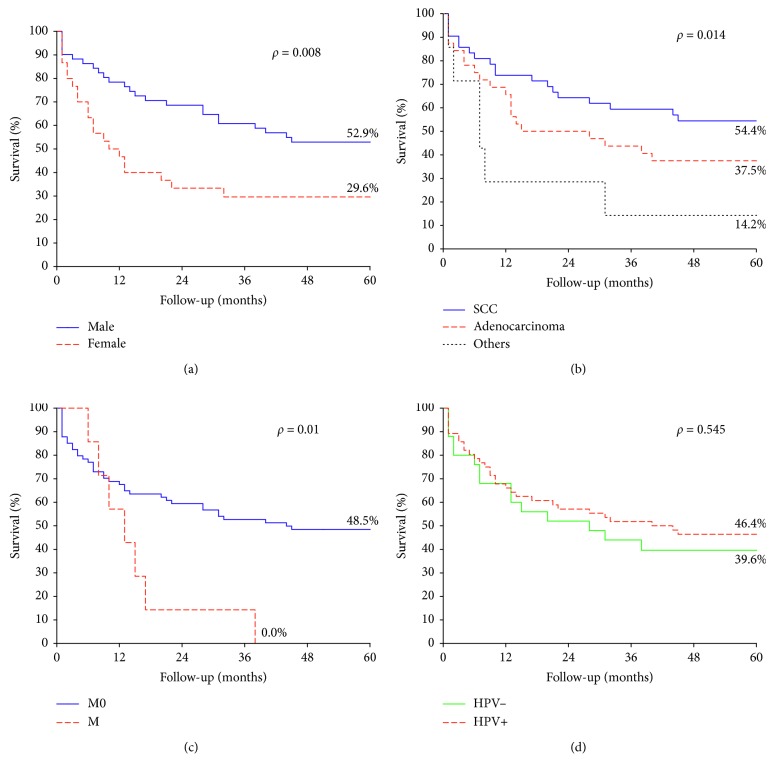
Survival curves for patients with anal cancer according to clinical and pathological characteristics. (a) Gender; (b) tumor histological type; (c) metastasis (M); (d) HPV detection. Others include basaloid carcinoma, neuroendocrine, and cloacogenic.

Overall survival at 60 months for patients with anal cancers was 44.3% (Figure 3). The mean follow-up was 31 months (±59.4) with a minimum of 1 month and a maximum of 191 months. The prognostic factors were being female (Figure 4(a)), squamous cell carcinoma (Figure 4(b)), and the presence of distant metastasis (Figure 4(c)). Survival was not influenced by the presence of HPV (Figure 4(d)), lymphatic dissemination (*p*=0.84), or tumor size (*p*=0.08); however, all individuals with T3 or larger tumors were deceased after 38 months.

## 4. Discussion

HPV was present in 69% of anal cancer samples, and HPV 16 was the most prevalent genotype (78.5%), followed by HPV 33 (10.7%) and HPV 18 (8.9%). Although the prevalence of HPV in the anal cancers evaluated was lower than the overall percentage reported in other studies [2, 8, 14, 15, 20–25], it is worth noting that this study included a relatively large number of anal adenocarcinomas (39.5%) and that there are few studies investigating the association of HPV with this histologic type of anal cancer [23–26]. SCC is the histological type most associated with the presence of HPV. In our study, the prevalence of the virus in this histological type was 88%, and HPV 16 was present in 82% of the genotyped samples. The high prevalence of HPV in anal SSC has also been observed in 15 other studies, in which the overall prevalence of HPV ranged from 60.6% to 100% [1, 2, 8, 14–16, 20–28]. These large number of SCC cases positive for HPV suggest that virus infection is a necessary cause for this type of anal cancer, as well as cervical cancer, mainly because the transition zone of the anal canal is very similar to the cervical squamocolumnar junction. Because HPV is a virus that is highly tropic in regions covered by squamous epithelium or high proliferative cell activity, these sites become more vulnerable to viral infection [6, 29].

Unlike SCC, adenocarcinoma of the anus is not intrinsically related to HPV infection. In some studies, this histological type is not analyzed because it is considered an extension of rectal cancer [30]. In this study, the adenocarcinomas included (39.5%) were classified histologically and clinically as primary anal cancers, of which 43.8% were infected by HPV, mainly by high-risk genotypes, such as HPV 16.

Samples from two individuals were classified as low-risk HPV (HPV 6 and HPV 11) during genotyping. These genotypes are responsible for papillomatous lesions and are not considered carcinogenic [29, 31]. It is complicated to define the association of cancer or a precursor lesion with a specific HPV genotype, since the molecular methods used in genotyping generally do not preserve the tissue architecture [22]. Moreover, the PCR method detects the HPV genome in the tumor but does not distinguish whether or not HPV is transcriptionally active, making it impossible to conclude which genotype(s) were actually involved in the carcinogenesis [1, 4, 17].

Multiple infections were observed in 14.3% of patients, a considerably higher value than in the other studies [25, 28, 32, 33]. The genotyping used in this study is based on the technique of reverse hybridization, which in this study identified a larger number of HPV genotypes and the presence of multiple infections. This method uses SPF10 primer oligonucleotides that are able to amplify a broad spectrum of HPV genotypes by virtue of their high sensitivity [2, 13, 32].

As expected, HPV 16 was the most prevalent genotype and was present in 100% of multiple infections. Genotype 16 is considered to be high risk and tends to persist in the host for a longer time [34]. Its oncogenic potential is related to its high expression of the viral oncoproteins E6 and E7, and its ability to integrate the viral genome into host cell DNA [29, 31]. Cell targets of HPV oncoproteins are primarily pRb and p53; however, underlying mechanisms associated with other cellular proteins may occur during carcinogenesis leading to cell cycle progression, evasion of apoptosis, DNA damage, and suppression of the immune response [35].

In the analyzed anal carcinomas, HPV 33 was the second most prevalent genotype (10.7%), followed by HPV 18 (8.92%). The presence of HPVs 16 and 18 is already well established, and both are targets of HPV prophylactic vaccines [13, 36–39]. In our research, only two samples showed both genotypes. The high prevalence of HPV 33 has been found before in other studies, ranging from 2 to 11.9% [20, 24, 25, 27] and suggests that the vaccine might have greater coverage if this genotype were included in the free vaccination program in developing countries such as Brazil, similar to, for example, the introduction of the nonavalent vaccine that includes the genotypes HPVs 16, 18, 31, 33, 45, 52, 58, 6, and 11 [36].

Regarding the analysis by gender, the prevalence of HPV in anal cancers was higher in women (78.4%), as observed in other studies [1, 2, 25, 32, 37]. Persistent long-term HPV infections or newly acquired infections may contribute to the increased susceptibility in women when associated with hormonal or immunological status changes [1, 2, 32, 37, 40].

The incidence of anal cancer increases with age, and the peak incidence occurs between 50 and 70 years with the mean age at diagnosis being 62 years [2, 32]. In this study, age ranged from 36 to 92 years, and the mean age at diagnosis was 61 years. Similar ages were reported in different studies [2, 13, 25, 26].

Overall survival at 60 months was 44.3%. In the United States, the five-year survival for anal cancer is higher, around 67% [41]. It is important to emphasize that Brazil is still a country with many socioeconomic problems, and access to health services, although free of charge, fails due to delays in patient services, and patients do not always have access to educational information for the prevention and treatment of diseases. In this way, anal cancers end up being stigmatized, surrounded by prejudices. In addition, their symptoms are very similar to common diseases of the anus, making their diagnoses neglected, and as a result, diagnoses are performed in more advanced stages and not always easy to treat [6, 42, 43].

Little is known about the prognostic importance of HPV in anal cancers, and the number of studies that deal with the relationship between survival and HPV infection in anal cancer is low [19, 20, 24, 34, 44]. The presence of the virus as a prognostic factor was investigated in this study; however, the results obtained did not allow a significant conclusion about these variables. Some studies have considered the presence of the virus as an important factor in the prognosis of anal cancer [19, 20, 34]. Biomarkers such as p16 have been investigated for prognostic use in anal intraepithelial neoplasias and anal carcinomas [20, 34, 44].

In this study, gender (*p*=0.008), histological type (*p*=0.01), and the presence of distant metastasis (*p*=0.01) were observed as prognostic factors. Women with anal cancer had a worse prognosis (29.6%) when compared with men (52.9%). These data reflect the need for follow-up of women, not only with the Pap smear, which is intended for the detection of cervical cancer, precancerous lesions, and other genital diseases but also the introduction of anuscopy as a screening method for the detection of anal cancer in women [43].

Regarding histological type, patients with anal adenocarcinoma had shorter survival compared to patients with SCC. Some studies have suggested that patients with anal adenocarcinoma have a worse prognosis, but these studies are limited by their sample size [37, 45]. Although the etiology of adenocarcinoma is very similar to that of SCC, it originates from the glandular tissue generally from the upper part of the anal canal, making it difficult to distinguish it from the adenocarcinoma of the lower rectum [24, 30, 45, 46]. The Franklin study (2016) has shown that survival in patients with anal and rectal adenocarcinomas is significantly worse than in those with anal SCC regardless of the type of treatment, suggesting that adenocarcinomas exhibit unique and aggressive behavior in relation to the site of other carcinomas [45].

It is unclear whether samples negative for HPV DNA are actually negative or whether these cases have actually been triggered by other carcinogens. At the molecular examination, only five of 42 SCC samples were negative for HPV. The rest of the negative anal cancers were adenocarcinomas or cloacogenic, basaloid, and neuroendocrine carcinomas.

The lack of some relevant information in the medical records limited the ability to investigate some variables, such as sexual behavior, which is known to increase the risk of HPV infection [14, 43, 47]. Even so, our data adequately represented the reality of individuals with anal cancer in the Midwestern region of Brazil, in the service area of this referral hospital.

The association of HPV to anal cancer has been demonstrated in this study, especially in SCC. However, the presence of HPV did not influence the prognosis of patients with anal cancer. The most prevalent genotypes were HPVs 16, 33, and 18. Further research on the role of HPV and its genotypes in anal carcinogenesis needs to be planned, and the prognostic aspects of anal cancer need to be better elucidated. Risk groups considered for anal cancer comprised mostly of HIV-positive individuals and men who have sex with men; however, as shown in this study, women need to be included in new public policies of this group.

## Figures and Tables

**Table 1:** Sociodemographic and clinical/pathological characteristics of patients with anal cancers (*n *81).

	*n*	%
Gender		
Female	51	63.0
Male	30	37.0
Age at diagnosis (years)		
<61 years	38	46.9
≥61 years	43	53.1
Marital status		
Single	37	45.7
Married	40	49.4
Ethnicity		
White	29	35.8
Brown (pardo)	48	59.3
Black	4	4.9
Smoker		
Yes	22	27.2
No	54	66.6
Alcohol consumption		
Yes	17	21.0
No	58	71.6
Tumor location		
Anal canal	59	72.8
Anal border	8	9.9
Both	14	17.3
Histological type		
SCC	42	51.9
Adenocarcinoma	32	39.5
Others	7	8.6
Treatment		
Surgery	65	80.2
Radiotherapy	62	76.5
Chemotherapy	51	63.0
No treatment	4	4.9
Size of tumor		
T1-2	58	71.6
T3-4	22	27.2
Not specified	1	1.2
Lymph node metastasis		
Yes	21	25.9
No	60	74.1
Distant metastasis		
Yes	7	8.6
No	74	91.4
Sites of distant metastasis		
Liver	2	2.5
Lung	2	2.5
Bladder	1	1.2
Uterus	1	1.2
Vagina	1	1.2
Death record		
Yes	45	55.6
No	36	44.4

SCC: squamous cell carcinoma. Others: basaloid carcinoma, neuroendocrine, and cloacogenic. Number of patients with data not informed: marital status 4 (4.9%); smoking 5 (6.2%); alcohol consumption 6 (7.4%); and not specified size of tumor 1 (1.2%).

**Table 2:** HPV DNA and anal cancers, according to sociodemographic and clinical/pathological characteristics.

Variables	HPV + (*n*)	%	HPV − (*n*)	%	*p*	OR (CI 95%)
Gender						
Female	40	78.4	11	21.6	0.01^*∗*^	3.18 (1.19–8.48)
Male	16	53.3	14	46.7		
Age at diagnosis (years)						
<61 years	26	68.4	12	31.6	0.89	0.93 (0.36–2.41)
≥61 years	30	69.8	13	30.2		
Marital status						
Single	24	64.9	13	35.1		
Married	29	72.5	11	27.5	0.46	0.70 (0.26–1.84)
Smoker						
Yes	15	68.2	7	31.8	0.97	0.98 (0.33–2.86)
No	37	68.5	17	31.5		
Alcohol consumption						
Yes	11	64.7	6	35.3	0.63	0.76 (0.24–2.39)
No	41	70.7	17	29.3		
Lesion location						
Anal canal	38	64.4	21	35.6	0.55	0.60 (0.11–3.26)
Anal border	6	75.0	2	25.0		
Both	12	85.7	2	14.3		
Histological type						
SCC	37	88.1	5	11.9	0.0001^*∗*^	9.51 (2.96–30.50)
Adenocarcinoma	14	43.8	18	56.2		
Others	5	71.4	2	28.6		
Size of tumor						
T1-2	41	70.6	17	29.3	0.44	1.50 (0.52–4.25)
T3-4	14	63.6	8	36.4		
Lymph node metastasis						
Yes	14	66.7	7	33.3	0.77	0.85 (0.29–2.48)
No	42	70.0	18	30.0		
Distant metastases						
Yes	5	71.4	2	28.6	1.00	1.13 (0.20–6.25)
No	51	68.9	23	31.1		
Death						
Yes	30	66.7	15	33.3	0.59	0.76 (0.29–2.00)
No	26	72.2	10	27.8		

SCC: squamous cell carcinoma. Others: basaloid carcinoma, neuroendocrine, and cloacogenic; number of patients with data not informed that they were positive for HPV: marital status 3; smoking 4; alcohol consumption 4; and size of tumor not specified 1. ^*∗*^Statistically significant values for *p* ≤ 0.05.

**Table 3:** Distribution of HPV genotypes according to histological type of anal carcinoma.

HPV	SCC (37/42)		Adenocarcinoma (14/32)		Other carcinomas (5/7)	
	*n*	%	*n*	%	*n*	%
HPV 16 only	25	67.6	9	64.3	2	40.0
HPV 16 and others	4	10.8	1	7.1	1	20.0
HPV 18 only	2	5.4	1	7.1	0	0.0
HPV 18 and others	0	0.0	0	0.0	0	0.0
HPV 16 and HPV 18	1	2.7	1	7.1	0	0.0
Others single HPV	5	13.5	2	14.3	2	40.0
HPV negative	5	11.9	18	56.3	2	28.6

Other HPV: HPV 6, 11, 33, 35, 39, 52, 56, 58, and 74. Other carcinomas: basaloid carcinoma, neuroendocrine, and cloacogenic.

## Data Availability

The medical records, histopathological reports, and protocols of analysis of the sample data used to support the findings of this study are available from the corresponding author upon request or are included within the article. For any additional information, request should be given to larisse.dalla@gmail.com.
